# A critical perspective of prion disease surveillance in Brazil

**DOI:** 10.3389/fnins.2026.1762241

**Published:** 2026-01-29

**Authors:** Breno José Alencar Pires Barbosa, Maria Luiza Vasconcelos Montenegro, José Eriton Gomes da Cunha

**Affiliations:** 1 Medical Science Center, Federal University of Pernambuco, Recife, Brazil; 2 Faculty of Medical Sciences, University of Pernambuco, Recife, Brazil; 3 National Prion Disease Pathology Surveillance Center, Case Western Reserve University, Cleveland, OH, United States

**Keywords:** Creutzfeldt-Jakob syndrome, dementia, neurocognitive disorders, prion diseases, public health surveillance

## Abstract

**Introduction:**

Prion diseases (PrD) are a group of rapidly progressive dementias. Among its subtypes, Creutzfeldt-Jakob Disease (CJD) is the most common, affecting around 1–2 individuals per million inhabitants yearly. In 2005, Brazil’s Ministry of Health (MH) initiated a surveillance program for CJD, creating a protocol to report the cases. Despite advances, the MH still struggles to make a reliable database to determine PrD profile in Brazil. Therefore, the aim of the present study was to understand the Brazilian PrD surveillance system.

**Methods:**

This is a retrospective and descriptive study based on the epidemiological records of CJD surveillance from 2005 to 2021 in the Ministry of Health’s Epidemiological Bulletin published in 2022.

**Results:**

1,576 suspected cases of CJD were reported, concentrated in the Southeast, South and Northeast regions of Brazil. Among the notifications, the following age groups predominated: 55–74 years (60.2%), 45–54 years (15%), and 75–85 years (11.8%). Suspected cases were mainly represented by women (53.6%), white individuals (61.4%), and residents of urban areas (90%). 547 cases (34.7%) were confirmed based on the following confirmation criteria: laboratory (65.6%), clinical-epidemiological (29.4%), and not informed (4.9%).

**Conclusion:**

In this period, the expected number of cases for the Brazilian population would be 3,200. Additionally to underreporting, the data shared by MH is limited due to the use of a database destined to mainly observe epidemic outbreaks and mismatches between official documents. Despite some progress since 2005, PrD surveillance in Brazil faces significant problems, due to the inaccurate treatment of these data and the lack of a specific database for CJD.

## 1 Introduction

Prion diseases (PrD) are a group of rare, rapidly progressive neurological conditions with no known effective treatment. The etiological agent responsible for PrD, known as PrPSc, consists of a conformational isomer of the prion protein (PrPc), which is naturally found on the cell surface of various cell types, including neurons (Sigurdson et al., 2019). Kuru, Gerstmann-Sträussler-Scheinker syndrome, Fatal Familial Insomnia, and Creutzfeldt-Jakob Disease (CJD) are different subtypes of PrD capable of infecting humans (Appleby et al., 2022). CJD is the most common and affects around 1–2 individuals per million inhabitants worldwide every year (Klug et al., 2013). CJD is classified as sporadic (sCJD), genetic (gCJD), and acquired, which includes iatrogenic (iCJD) and new variant CJD (vCJD) (Geschwind, 2015).

The definitive diagnosis of CJD can only be made post-mortem based on the histopathological study of a brain tissue sample (Kretzschmar and Feiden, 2002). However, other tests are essential to reinforce the diagnostic hypothesis, such as magnetic resonance imaging (MRI) and genetic testing (Araújo, 2013). Electroencephalography (EEG) and cerebrospinal fluid (CSF) studies using the real-time quaking-induced conversion (RT-QuIC) technique or the identification of the 14-3-3 protein are also helpful during the diagnostic investigation process (Rhoads et al., 2020).

Following the vCJD crisis and its association with contaminated cattle herds, several countries established surveillance systems primarily to monitor this disease subtype (Anderson et al., 1996). In 2005, the Brazilian Ministry of Health (MH) included CJD on the national list of compulsory notification of diseases, illnesses, and public health events (Martins et al., 2007), and 603 suspected CJD cases were reported up to June 2018. Of these, 404 were undiagnosed (Cardoso et al., 2015; Ministry of Health, 2018). Difficulties in performing imaging examinations and neuropathological analyses are found in several medical units in Brazil, making diagnosis problematic. Initiatives to make RT-QuIC available are still modest, and some diagnosed patients had samples sent to centers outside the country for biomarker analysis, allowing for more excellent coverage of the diagnostic criteria (Barbosa et al., 2020).

Despite the progress made, the epidemiological profile of CJD in Brazil created by MH still has debilities, mainly because of the misuse of the CJD datasheet, the lack of a specific database focused on the critical information for that disease, and inconsistent data when compared to information released by MH in different documents for the same period. Given the importance of rigorously monitoring the behavior of PrD in Brazil, as this is a critical disease for public health, new research should be dedicated to studying the subject. Therefore, this study aimed to evaluate the Brazilian PrD surveillance system, highlighting some of its weaknesses and pointing out prospects for making it more reliable and efficient.

## 2 Materials and methods

### 2.1 Study design

This is a retrospective and descriptive study based on a comprehensive analysis of epidemiological records from 2005 to 2021 from CJD surveillance in the MH’s Epidemiological Bulletin, published in 2022 (Ministério da Saúde, 2022). The CJD Notification and Investigation Protocol created by the Ministry of Health (2018) and data from the Information Access Law were also analyzed.

### 2.2 Sample

The information used to compose this study is available in the MH’s Epidemiological Bulletin on CJD surveillance published in 2022, which used data from the Department of Informatics of the Ministry of Health/Notifiable Diseases Information System (DATASUS/SINAN), an online platform with free public access. The Notifiable Diseases Information System (SINAN), regulated in 1988, is a national database created to gather notifications and investigations of cases of diseases belonging to the list of mandatory reporting, such as CJD, tuberculosis, and botulism in Brazil, and, for that reason, it was used as the primary data source for this research. We reviewed notifications of suspected cases of CJD from January 2005 to December 2021.

We analyzed the following sociodemographic variables: age, sex, race, area of residence, and state of residence. In turn, the clinical-epidemiological variables included were signs and symptoms, final classification, confirmation criteria, evolution of the case, death, and federation unit of notification. Cases that did not register the date of onset of symptoms were excluded from the investigation.

### 2.3 Classification of CJD cases and epidemiological notification process in Brazil

In identifying a suspected case of CJD, it is recommended that the health professional make the notification using the Creutzfeldt-Jakob Disease investigation form, which must be forwarded to the state epidemiological surveillance where the case was suspected, and, subsequently, to the General Coordination of Transmissible Diseases of the Ministry of Health. The health professional must also complete, within 7 days of notification of the case, the SINAN notification/conclusion form at the health unit where the case was identified (Ministry of Health, 2018).

The investigation of a suspected case of CJD occurs through the analysis of the clinical picture presented by the patient and epidemiological risk factors, such as a history of similar cases in the family, a history of neurosurgeries, and international travel to countries with reports of the bovine form of the disease. A suspected case is defined as progressive dementia over a 2-years period with at least two of the following characteristics: pyramidal or extrapyramidal signs; myoclonus; visual or cerebellar changes; akinetic mutism; presence or absence of psychiatric symptoms at the onset of the condition; dysesthesias and painful sensory symptoms. Before the post-mortem necropsy, the clinical investigation includes EEG, MRI, and laboratory tests, such as the identification of the 14-3-3 protein in the cerebrospinal fluid and genetic tests in search of mutations (Ministry of Health, 2018).

The suspected case can be classified as possible if the EEG is not typical or has not been performed, or probable if the EEG shows a pattern characteristic of the disease, the cerebrospinal fluid examination is positive for the 14-3-3 protein, and death occurs in less than 2 years. Confirmation of the case requires a standard neuropathological examination, identification of the protease-resistant prion protein, and/or the presence of fibrils compatible with PrPSc. It is also important to recognize the PrPSc mutation in genetic cases and to identify neurosurgical risk factors in iatrogenic cases (Ministry of Health, 2018).

### 2.4 Ethical considerations

As this study used only publicly accessible secondary data, the identities of the subjects involved were not disclosed, so approval by the local Research Ethics Committee was not necessary.

## 3 Results

Between 2005 and 2021, SINAN recorded 1.576 notifications of suspected cases of CJD, which were concentrated in the Southeast, South, and Northeast regions of Brazil, especially in the states of São Paulo (32.5%), Paraná (12%), and Minas Gerais (10%). The 55–74 age group predominated (60.2%), followed by 45–54 (15%) and 75–85 (11.8%).

The years with the highest number of notifications were 2019 (11%), 2021 (9%), and 2014 (9%). The age range with the fewest notifications was 0–24 years (1.7%). In addition, most notified cases occurred among women (53.6%), white individuals (61.4%), and urban dwellers (90%) (Table 1).

**TABLE 1:** Sociodemographic characteristics of CJD suspected cases (*n* = 1.576) between 2005 and 2021.

Patient characteristics	*N*	%
**Gender**		
Male	730	46.3
Female	845	53.6
Ignored	1	0.1
**Race**		
White	968	61.4
Black	55	3.5
Yellow	27	1.7
Brown	349	22.1
Indigenous	–	–
Ignored	177	11.2
**Age (years)**		
0–24	27	1.7
25–34	30	1.9
35–44	92	5.8
45–54	237	15
55–64	476	30.2
65–74	473	30
75–85	186	11.8
>85	38	2.4
Not reported	17	1.1
**Residence zone**		
Urban	1,418	90
Rural	87	5.5
Peri-urban	7	0.4
Ignored	15	1
Not reported	49	3.1

*n*, sample size. Source: adapted from CJD Epidemiological Bulletin from the Ministry of Health, volume 53, number 39, 2022.

Of all the notifications, 547 cases (34.7%) were confirmed based on the following confirmation criteria: laboratory (65.6%), clinical-epidemiological (29.4%), and not informed (4.9%). Just five Brazilian states reported accurate information: Rondônia, Acre, Mato Grosso do Sul, Rio de Janeiro, and Rio Grande do Sul. MH’s Epidemiological Bulletin does not specify which laboratory tests or clinical-epidemiological criteria were used to confirm the cases, which hinders the evaluation of the data presented. On the other hand, in the CJD notification and investigation protocol, the MH cites the clinical and laboratory criteria for classifying a case as possible, probable, and definitive (Ministry of Health, 2018).

**FIGURE 1:**
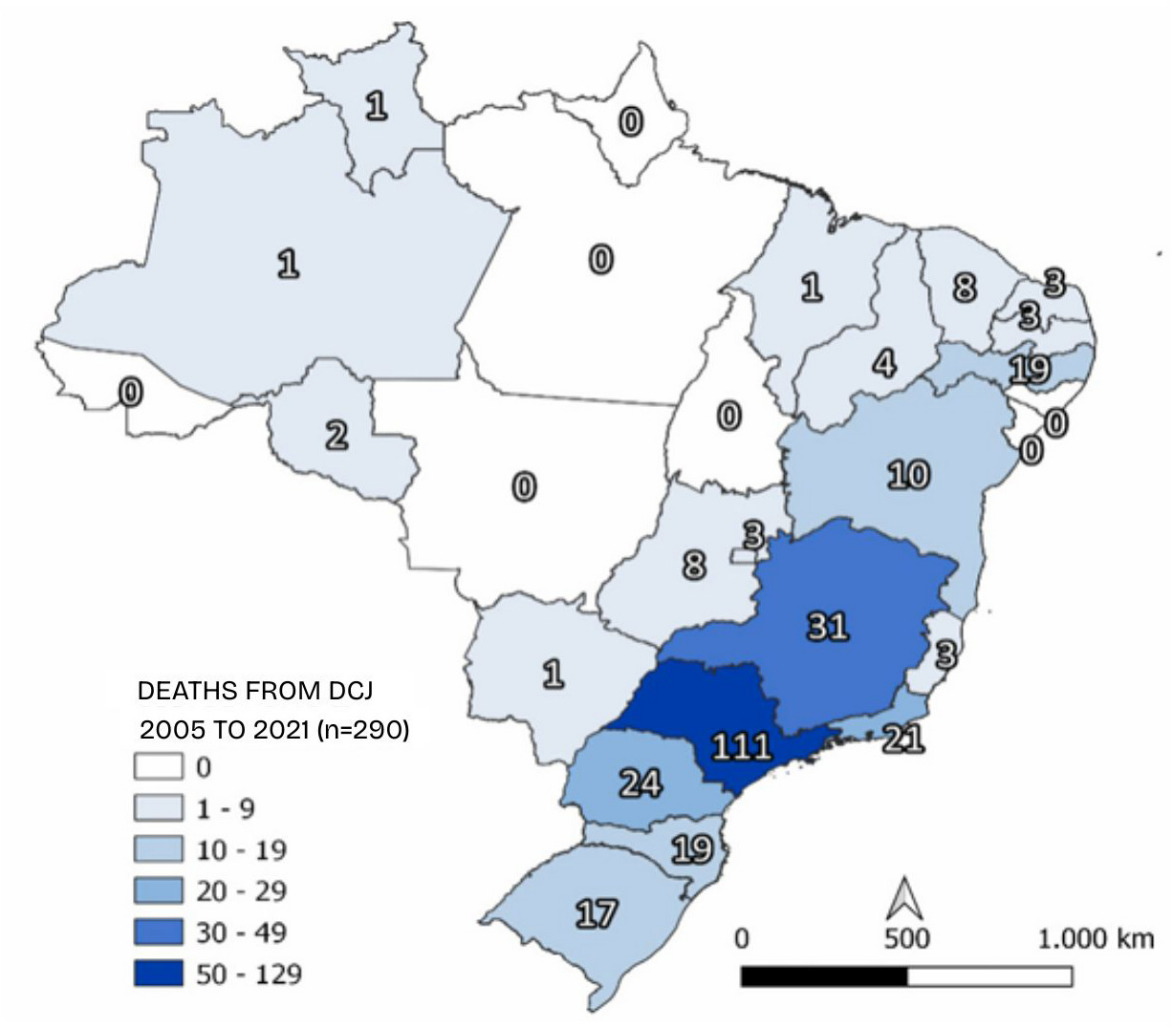
Geographic distribution of deaths from suspected cases of CJD in Brazil between 2005 and 2021. *n*, sample size. Source: adapted from CJD Epidemiological Bulletin from the Ministry of Health, volume 53, number 39, 2022.

It should be noted that the same document also states that only 18.4% of notified cases died from the disease and that 13.1% were cured, which is unexpected for the natural history of the disease, considering the 100% mortality rate of CJD already well established in current literature. In addition, 55.8% of the notifications did not provide data on how the cases evolved, and crucial information, such as the time interval from the onset of symptoms to diagnosis, was also not clarified in the document. Deaths were also more prevalent in São Paulo (38.3%), Minas Gerais (10.7%), and Paraná (8.3%) (Figure 1). Most deaths were recorded in the 65–74 age group (34.1%), followed by 55–64 (27.5%) and finally by 45–54 (14.4%) (Table 2). The highest deaths were in 2014 (12%) and 2016 (11.7%), respectively. Moreover, the information displayed in the tables differs grossly from that found in the text of this document, maybe due to how the raw data was exported from different software.

**TABLE 2:** Classification of cases (*n* = 1.576) and deaths (*n* = 290) suspected of CJD by age between 2005 and 2021.

Age (years)	Cases (*n* = 1.576)	Deaths (*n* = 290)
0–24	27	5
25–34	30	4
35–44	92	17
45–54	237	42
55–64	476	80
65–74	473	99
75–85	186	28
>85	38	6
Not reported	17	9

*n*, sample size. Source: adapted from CJD Epidemiological Bulletin from the Ministry of Health, volume 53, number 39, 2022.

## 4 Discussion

Only 547 of the 1,576 cases of CJD reported as suspected by the MH between 2005 and 2021 were confirmed. However, considering the estimates in the literature and the Brazilian population for this period, the total number of reported cases should have been close to 3,200, exposing an intense underreporting of this condition (Klug et al., 2013).

We observed that data on PrD in the national territory are still organized in a way that falls short of expectations for creating a rigorous surveillance system for this disease. Among the inconsistencies found in the MH’s epidemiological bulletin of CJD, there needs to be more detail regarding the method used to confirm cases, and there is a deficient description of how the condition evolved in 55.8% of notifications.

The sCJD usually affects older adults, with an average age of 67. On the other hand, cases of vCJD tend to occur in the younger population, with an average age of 26 (National Centre Research & Surveillance Unit, 2016). In this study, most of the notifications of suspected cases occurred in the 55–74 age group. However, the CJD Epidemiological Bulletin from the MH does not refer to the disease subtypes in the data presented, making it difficult to establish an accurate epidemiological profile.

There was also a predominance of females (53.6%) among the reported cases. This finding aligns with a prospective study in Japan, which found a prevalence of 58.1% of women in the sample of patients diagnosed with prion diseases (Nozaki et al., 2010). Concerning race, most of the cases were reported in white Brazilians (61.4%). Other studies in the literature have also reported a higher incidence of PrD in white individuals, which could be explained either by a possible genetic predisposition that has not yet been discovered in this part of the population (Sánchez-González et al., 2020; Tam et al., 2023) or by a notification bias since most reports describe data from high-income, Caucasian-predominant countries.

The Southeast, South, and Northeast were the Brazilian regions with the highest number of suspected cases of CJD between 2005 and 2021. Despite an 11-years gap, these exact locations also occupied the podium for PrD deaths recorded between 2005 and 2010 (Cardoso et al., 2015). This finding shows that these regions could have a more structured epidemiological surveillance system than other regions of the country.

Although diagnostic confirmation can only be made after the patient’s death, some procedures can be carried out while the patient is still alive during the investigative process. Real-time quaking-induced conversion (RT-QuIC), a method that amplifies the pathogenic prion protein in cerebrospinal fluid, has a sensitivity of 90.3% and specificity of 98.5% for CJD (Rhoads et al., 2020). This method is primarily unavailable in Brazil, despite some efforts, such as the report by Barbosa et al. (2020). The authors performed second-generation RT-QuIC in CSF samples from eight patients with suspected CJD and used patients with other neurological conditions as negative controls. Five of seven suspected cases had positive tests; two showed inconclusive results. Among controls, there was one false positive. The presence of the 14-3-3 protein in CSF has good sensitivity in diagnosing CJD, especially in sporadic forms, but this marker has been considered with variable specificity and sensitivity (Satoh et al., 2006).

Data from the CJD Epidemiological Bulletin from the MH suggests that some of the cases were confirmed through clinical aspects, which goes against the scientific premise that PrD can only be definitively diagnosed through histopathological examination. Considering the relevance of MRI in the diagnostic investigation of CJD, this procedure should be more emphasized in the CJD Notification and Investigation Protocol created by Ministry of Health (2018). Furthermore, this same document should define more clearly what constitutes a suspected case of CJD and what criteria are essential for confirmation, since the way it is presented is ambiguous and could cause confusion among non-specialists, because, for example, it does not specify which MRI sequences should be followed to obtain the images.

Unlike Brazil, the United Kingdom has the UK National CJD Research and Surveillance Unit, which is exclusively responsible for registering and monitoring suspected cases of CJD in the country. This is a major advancement, as it allows for the concentration of efforts directed toward surveillance and the creation of more effective diagnostic methods for CJD (Tam et al., 2023). Similarly, in the United States, there is the National Prion Disease Pathology Surveillance Center, which is supported by the US Centers for Disease Control and Prevention, and provides accurate diagnostic tests, such as RT-QuIC, to enable the rapid diagnosis of suspected cases (Sánchez-González et al., 2020).

In 1992, France created the National Surveillance Network of Transmissible Spongiform Encephalopathies, to which laboratories notify suspected cases of CJD through tests, such as the identification of the 14-3-3 protein in the CSF, and which allows physicians to directly contact neurologists to ensure the correct management of cases (Denouel et al., 2023). This model of an organized network of prion disease specialists who can be contacted directly by the attending physician of suspected cases makes the notification and follow-up process much more efficient. In Germany, the National Reference Center for Transmissible Spongiform Encephalopathies, which is linked to the German Ministry of Health, classifies suspected cases of CJD based on contact with health authorities where the case was identified (Hermann et al., 2023).

Despite being a notifiable disease with a high associated mortality rate, CJD surveillance in Brazil is still marked by underreporting of cases. This reality is alarming because, without understanding the real number of cases affecting Brazilians, it becomes unlikely to draw a reliable epidemiological profile of the population groups at highest risk for developing the disease. It is also worth noting that underreporting directly affects the creation of national policies for the prevention and control of the disease, since public health managers are guided by this same epidemiological surveillance data. We believe we can learn from other referral centers, like in the USA, Germany, and others, to create more reliable surveillance for CJD in Brazil.

Retrospective methodological design based on a public database is an important limitation of the study. Studies conducted with secondary data are subject not only to incorrect data entry but also to incomplete data, which favors the persistence of underreporting and significantly interferes with the quality of the data presented. Although SINAN is a government database, the record of cases that progressed to a cure is inconsistent with the prognosis of the disease, exposing biases in the accuracy of the data. In addition, some of the notifications did not contain the evolution of the cases, which reveals that the information made available through SINAN is still very superficial; therefore, although it is a useful tool for monitoring epidemiological outbreaks of certain diseases, the applicability of SINAN in the epidemiological surveillance of CJD, which has a long incubation period, is not ideal.

This document suggests, considering all the points highlighted, a review of how data on the epidemiology of CJD in Brazil are recorded and published, so that this study serves as a foundation for conducting prospective collaborative studies with other research centers, aligning Brazil with international epidemiological surveillance.

## 5 Conclusion

We propose that MH and other players, such as researchers, epidemiologists, and neurologists, create a specific PrD database using the CJD report form as the input data, not only the SINAN inputs. Also, MH should review the current protocol, adding more information about differential diagnosis, biomarkers such as RT-QuIC and MRI criteria. Additionally, patients and neurologists must have access to a comprehensive diagnostic protocol (e.g., MRI, CSF, and genetic studies) as a strategy to reduce disparities in care and data collection. All efforts should be pursued to create a comprehensive surveillance system that supports public health authorities in observing trends in CJD in Brazil.

## Data Availability

The original contributions presented in this study are included in this article/supplementary material, further inquiries can be directed to the corresponding author.
